# Meta-analyses of the relationship between five *CXCL8* gene polymorphisms and overall cancer risk, and a case-control study of oral cancer

**DOI:** 10.1186/s12903-024-04330-6

**Published:** 2024-05-28

**Authors:** Jie Peng, Yina Wang, Dan Kuang, Ying Wang, Gang Wu, Huangjing Li, Dan Li, Hong Cao

**Affiliations:** 1 Wuxi School of Medicine, Jiangnan University, Wuxi, 214000 China; 2 Affiliated Hospital of Jiangnan University, Wuxi, 214000 China; 3 Department of Stomatology, Nursing Department, Affiliated Hospital of Jiangnan University, Wuxi, 214000 China; 4 Department of Pathophysiology, Zhongshan School of Medicine, Sun Yat-Sen University, Guangzhou, 510080 China; 5 Department of Endocrinology, Affiliated Hospital of Jiangnan University, No. 1000 Hefeng Road, Binhu District, Wuxi, 214000 China

**Keywords:** CXCL8, Polymorphism, Cancer, Oral cancer, Meta-analysis, Risk

## Abstract

**Background:**

C-X-C motif chemokine ligand (CXCL8), also known as interleukin-8, is a prototypical CXC family chemokine bearing a glutamic acid-leucine-arginine (ELR) motif that plays key roles in the onset and progression of a range of cancers in humans. Many prior studies have focused on exploring the relationship between *CXCL8* gene polymorphisms and the risk of cancer. However, the statistical power of many of these reports was limited, yielding ambiguous or conflicting results in many cases.

**Methods:**

Accordingly, the PubMed, Wanfang, Scopus and Web of Science databases were searched for articles published until July 20, 2023 using the keywords ‘IL-8’ or ‘interleukin-8’ or ‘CXCL8’, ‘polymorphism’ and ‘cancer’ or ‘tumor’. Odds ratios (ORs) and 95% confidence intervals (CIs) were utilized to examine the association. The CXCL8 +781 polymorphism genotypes were assessed with a TaqMan assay.

**Results:**

About 29 related publications was conducted in an effort to better understand the association between these polymorphisms and disease risk. The *CXCL8 -*353A/T polymorphism was associated with an increased overall cancer risk [A vs. T, odds ratio (OR) = 1.255, 95% confidence interval (CI) (1.079–1.459), *P*_heterogeneity_ = 0.449, *P* = 0.003]. The *CXCL8* +781 T/C allele was similarly associated with a higher risk of cancer among Caucasians [TT vs. TC + CC, OR = 1.320, 95%CI (1.046–1.666), *P*_heterogeneity_ = 0.375, *P* = 0.019]. Furthermore, oral cancer patients carrying the *CXCL8* +781 TT + TC genotypes exhibited pronounced increases in serum levels of CXCL8 as compared to the CC genotype (*P* < 0.01), and also shown similar trend as compared to genotype-matched normal controls (*P* < 0.01). Finally, several limitations, such as the potential for publication bias or heterogeneity among the included studies should be paid attention.

**Conclusion:**

Current study suggested that the *CXCL8* -353 and +781 polymorphisms may be associated with a greater risk of cancer, which might impact cancer prevention, diagnosis, or treatment through the different expression of CXCL8. At the same time, the +781 polymorphism may further offer value as a biomarker that can aid in the early identification and prognostic evaluation of oral cancer.

**Supplementary Information:**

The online version contains supplementary material available at 10.1186/s12903-024-04330-6.

## Introduction

Over the past five decades, there have been profound achievements in the field of cancer research that have spurred the design of new analytical technologies, enabling details genetic studies that have provided nuanced insights into how best to detect, monitor, and treat affected patients. Although overall survival rates for many cancers have improved, the global burden of cancer continues to grow with a predicted 57% increase in incidence by 2040 that will translate to an approximately 64% increase in mortality rates [1–3]. Given the growing prevalence of many cancers, GLOBOCAN data estimates that in 2030, there will be over 24 million new cases of cancer globally, with almost 13 million deaths [4]. Meanwhile, cancer is predicted to surpass cardiovascular disease as the most common cause of death in the USA by the year 2030, killing an estimated 640,000 Americans per year [5].

Cancers are etiologically complex and shaped by a range of internal and external factors including genetics, endocrine activity, external environmental factors, body mass index (BMI), alcohol intake, and smoking history. Oncogenesis, tumor progression, and metastatic dissemination are highly dependent on the ability of tumors to establish an environment that is conducive to angiogenesis [6].

Previous studies have reported that approximately 70% of cancers are caused by somatic genetic polymorphisms that result from aging [7], at the same period, it is considered that environmental carcinogens can cause 70–95% of human cancer [8, 9], which show gene-environment interactions are popular in the development of cancer. Because the concept of epigenetics greys the boundary on this debate by clarifying how environmental factors such as climate, nutrition, stress, and toxicants influence gene expression, and thus biological processes, without altering the underlying DNA sequence [10]. DNA methylation (DNAm) at cytosine residues, histone tail modifications, chromatin architecture, and non-coding RNA constitute reversible epigenetic modifications involved in modulating gene expression [11, 12]. Localized angiogenic activity can be driven by the overproduction of pro-angiogenic factors relative to angiogenic inhibitors. Members of the CXC family of chemokines are cytokines that play particularly important roles as regulators of angiogenesis, serving as potent inhibitors or drivers of this process [6].

These differences in the angiogenic potential of CXC family chemokines are primarily related to the presence of the N-terminal Glu-Leu-Arg (ELR) motif, which is found in angiogenic member of this family [including C-X-C motif chemokine ligand 1 (CXCL1), CXCL2, CXCL3, CXCL5, CXCL6, CXCL7, and CXCL8] [13–15], whereas it is absent from not angiostatic family members (including CXCL4, CXCL9, CXCL10, and CXCL11) [16].

ELR+ CXC chemokines serve as important regulators of the growth and progression of a range of cancers [17]. Of these, CXCL8 was first identified as a chemoattractant for leukocytes [18], and it has since also been shown to promote angiogenesis and proliferation [19, 20]. There is growing evidence supporting a role for CXCL8 in the development of cancer [21]. Indeed, higher levels of CXCL8 have been linked to the progression and recurrence of breast, prostate, gastric, oral and lung cancers [22–26].

Cytokine gene promoters harbor polymorphisms that can impact the production of these cytokines [27]. CXCL8 is encoded by the *CXCL8* gene, which consists of a proximal promoter, four exons, and three introns present on chromosome 4q13-21 [22–24, 28]. Multiple *CXCL8* polymorphisms have been documented to date, with strong evidence related to polymorphisms at the -251 site [29]. Studies of polymorphisms at other sites (+781 rs2227306, -353 rs1454941, +678 rs7374124, +1633 rs2227543, +2767 rs1126647), however, have not been as in-depth. In addition, no meta-analysis has not been reported, so it is necessary and make sense to perform a comprehensive analysis to obtain a convince conclusion. The present study was thus developed with the goal of conducting pooled analyses of all case-control studies focused on above five polymorphisms in order to generate stronger evidence whether significant associations were existed. Furthermore, based on my own Department and diagnosed patients, we explored the relation between +781 polymorphism and the clinical features of oral cancer to define novel biomarkers, differences in CXCL8 levels were compared between patients with oral cancer and healthy controls as a function of *CXCL8* +781 genotype.

## Materials and methods

### Study selection and data extraction

Initially, the PubMed, Wanfang, Scopus and Web of Science databases were searched for articles published as of July 20, 2023 using the keywords ‘IL-8’ or ‘interleukin-8’ or ‘CXCL8’, ‘polymorphism’ and ‘cancer’ or ‘tumor’. No language or publication year restrictions were imposed on the search. The references of retrieved articles and reviews were additionally manually searched for relevant studies. Eligible studies were those that: (a) evaluated correlations between cancer risk and one or more of the selected polymorphisms, (b) were case-control studies, (c) included age- and gender-matched control groups, and (d) had an available full-text manuscript. Studies were excluded if they: (a) lacked a control population, (b) did not provide genotype frequencies, (c)were duplicate studies, or (d) exhibited clear evidence of bias. Literature search results were reviewed by two investigators. Collected data from identified studies included first author, publication year, country, ethnicity, cancer type, genotypes in the case and control groups, source of controls, HWE analyses of controls, and genotyping methods (PCR-RFLP, PCR-SSP, PCR-ARMS, PCR-AS, real-time PCR, TaqMan).

### Statistical analyses

Odds ratios (ORs) and 95% confidence intervals (CIs) were utilized to examine the association between *CXCL8* polymorphisms and the risk of cancer based on genotypic frequency levels in cases and control subjects. Subgroup analyses were initially conducted stratified according to cancer type. Any cancers for which only one study was available were pooled under the category of “other cancers”. Ethnicity was classified as Caucasian, African, or Asian. Subgroup analyses were also conducted based on the source of control subjects, separately assessing population-based (PB) and hospital-based (HB) studies.

Pooled OR significance was assessed using the Z-test [30]. Chi-square-based Q tests were used to assess heterogeneity, with *P* < 0.05 being indicative of significant heterogeneity, in which case pooled ORs were analyzed with a random-effects model (DerSimonian and Laird method), whereas a fixed-effects model (Mantel–Haenszel method) was otherwise employed [31, 32]. For the +781, -353, +678, +1633, +2767 polymorphisms in the *CXCL8* gene, associations between genotype and cancer risk were assessed using dominant (MM + MW vs. WW), heterozygote comparison (MW vs. WW), allelic contrast (M-allele vs. W-allele), homozygote comparison (MM vs. WW), and recessive (MM vs. MW + WW) genetic models. Begg’s funnel plot, Egger’s test, Trim and Fill model were used to evaluate funnel plot asymmetry to detect publication bias [33], with *P* < 0.05 as the cut-off to define significance. The Pearson chi-square test for goodness of fit was used to detect departures from Hardy-Weinberg equilibrium (HWE) with respect to the frequencies of *CXCL8* polymorphisms, using *P* < 0.05 as the cut-off to define significance. Stata v11.0 (StataCorp LP, TX, USA) was used to conduct statistical analyses.

### Bioinformatics analyses

CXCL8 expression in most tumor types and paracancerous tissues were assessed with the GEPIA (http://gepia.cancer-pku.cn/) and UALCAN (https://ualcan.path.uab.edu/analysis.html) databases.

### Genotyping

CXCL8 +781 polymorphism genotyping has been performed with a range of techniques across studies, including qPCR, Taqman, amplification refractory mutation system-PCR, and restriction fragment length polymorphism PCR approaches. For the present study, CXCL8 +781 polymorphism genotypes were assessed with a TaqMan assay using the approach documented by Castro et al. [34].

### Study population

In total, this study enrolled 85 patients from the Affiliated Hospital of Jiangnan University who were newly diagnosed with oral cancer from April 1, 2020 – September 1, 2022. All patients had pathologically confirmed oral cancer diagnoses as determined by pathologists from the Department of Pathology of the Affiliated Hospital of Jiangnan University. An age-matched healthy control group (*n* = 85) was additionally recruited during this same time period from among individuals undergoing routine physical examinations. The exposure information of betel quid chewing, smoking and drinking were obtained by questionnaire, and medical information of cases was obtained from medical records, including TNM clinical stage, primary tumor size, lymph node metastasis and histological grade. According to the seventh edition of the American Joint Committee on Cancer (AJCC) staging manual, oral cancer patients were classified according to clinically TNM staging system. Tumor differentiation was examined by pathologists also according to the AJCC classification. All participants provided 3 mL samples of peripheral blood. This study was approved by the Institution Review Board of the Affiliated Hospital of Jiangnan University, and all patients provided written informed consent prior to sample collection (the ethical code: LS202128).

### ELISA assay

Blood samples were collected in angicoagulant-free tubes, after which serum separator tubes (SSTs) were utilized and samples were allowed to clot overnight at 4 °C or at room temperature for 2 h. Samples were then centrifuged (1000 × g, 15 min), after which serum was collected and immediately assessed or stored at -20 °C or -80 °C for future analyses, minimizing repeated freezing and thawing. Serum CXCL8 levels were detected with an ELISA kit (Abcam Co. Ltd.). Absorbance at 450 nm was assessed, with correction at 540 or 570 nm. For further details, see the manufacturer’s website (https://www.abcam.cn/products/elisa/human-il-8-elisa-kit-ab214030.html).

## Results

### Study selection

**Fig. 1:**
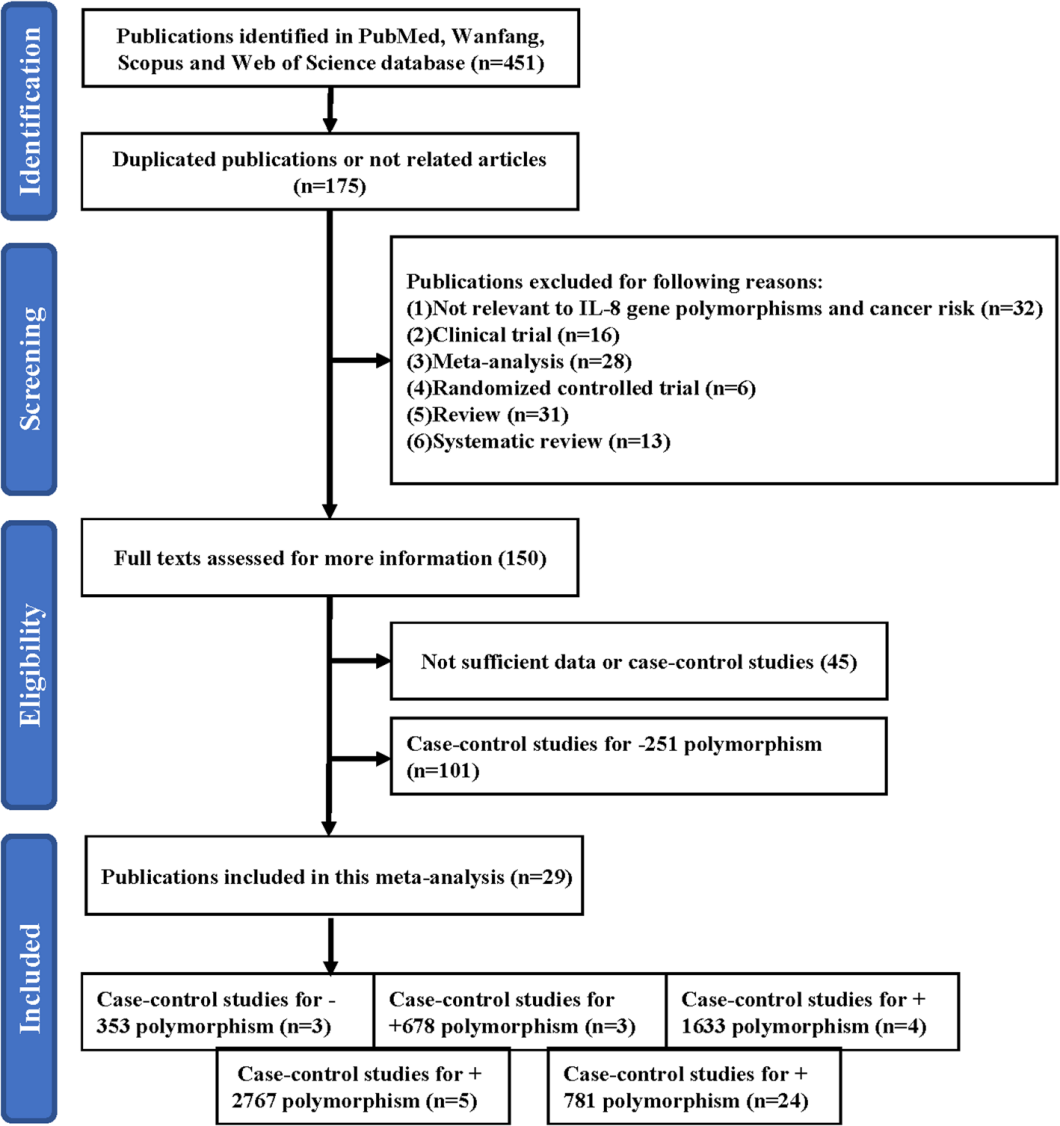
Flow chart outlining the study selection process used to identify the 28 case-control studies eligible for inclusion in this meta-analysis

An initial literature search identified 451 potentially relevant articles, for which 175 were duplicates, 126 were excluded because they were unrelated to the association between *CXCL8* polymorphisms and the risk of cancer (*n* = 32), clinical trials (*n* = 16), meta-analyses (*n* = 28), randomized controlled trials (*n* = 6), reviews (*n* = 31), systematic reviews (*n* = 13), or lacked sufficient data as case-control studies (*n* = 45). An additional 101 case-control studies focused on the *CXCL8* -251 site polymorphism were excluded because this polymorphism has been widely reported the association with several kinds of cancer risk through meta-analysis [35–37]. The remaining 29 studies were incorporated into the present meta-analysis (Fig. 1). The characteristics of these case-control studies are summarized in Table 1, and they included 3 studies focused on the -353 site, 3 related to the +678 site, 4 related to the +1633 site, 5 related to the +2767 site, and 24 related to the +781 site (Fig. 1).

**Table 1:** Characteristics about polymorphisms in CXCL8 gene polymorphisms and cancer risk for included studies

Author	Year	Country	Ethnicity	Cancer type	Case	Control	SOC	Case			Control			HWE	Method
-353 rs1454941689								AA	AT	TT	AA	AT	TT		
Wei	2007	China	Asian	nasopharyngeal carcinoma	280	290	PB	55	139	86	47	131	112	0.406	PCR-RFLP
Wang	2014	China	Asian	hepatocellular carcinoma	205	208	HB	37	95	73	34	94	80	0.474	PCR-RFLP/PCR-SSP
Zhang	2017	China	Asian	breast cancer	442	447	HB	358	67	17	337	82	28	< 0.001	PCR-RFLP
+678rs73741247								TT	TC	CC	TT	TC	CC		
Wei	2007	China	Asian	nasopharyngeal carcinoma	280	290	PB	38	104	138	35	111	144	0.064	PCR-RFLP
Ahirwar	2010	India	Asian	bladder cancer	205	270	PB	24	32	149	22	61	187	< 0.001	AS-PCR
Wang	2014	China	Asian	hepatocellular carcinoma	205	208	HB	27	78	100	26	82	100	0.161	AS-PCR
+1633rs2227543								TT	TC	CC	TT	TC	CC		
Chien	2011	China	Asian	hepatocellular carcinoma	131	340	HB	16	58	57	59	159	122	0.562	PCR-RFLP
Liu	2012	China	Asian	oral cancer	270	350	HB	44	126	100	61	164	125	0.569	PCR-RFLP
Koensgen	2014	Germany	Caucasian	ovarian cancer	246	62	HB	61	123	62	11	31	20	0.865	PCR-RFLP
Huang	2018	China	Asian	nasopharyngeal carcinoma	176	352	HB	35	83	58	73	158	121	0.109	PCR-RFLP
+2767rs1126647								TT	TA	AA	TT	TA	AA		
Chien	2011	China	Asian	hepatocellular carcinoma	131	340	HB	16	55	60	59	156	125	0.392	PCR-RFLP
Liu	2012	China	Asian	oral cancer	270	350	HB	43	123	104	61	161	128	0.029	PCR-RFLP
Koensgen	2014	Germany	Caucasian	ovarian cancer	268	426	HB	57	124	87	61	228	137	0.029	PCR-RFLP
Hsieh	2007	China	Asian	leiomyoma	162	156	HB	23	71	68	32	65	59	0.078	PCR-RFLP
Huang	2018	China	Asian	nasopharyngeal carcinoma	176	352	HB	32	84	60	75	149	128	0.012	PCR-RFLP
+781rs2227306								TT	TC	CC	TT	TC	CC		
Liu	2015	China	Asian	glioma	300	300	HB	37	97	166	31	108	161	0.049	PCR-RFLP
Kamangar	2006	USA	Caucasian	gastric cancer	111	208	PB	12	52	47	22	105	81	0.158	TaqMan
Bo	2010	China	Asian	gastric cancer	208	190	HB	39	99	70	32	82	76	0.225	PCR-RFLP
Chien	2011	China	Asian	hepatocellular carcinoma	131	340	HB	9	57	65	50	164	126	0.776	PCR-RFLP
Liu	2012	China	Asian	oral cancer	270	350	HB	35	118	117	52	169	129	0.781	PCR-RFLP
Qin	2012	China	Asian	oral cancer	150	150	HB	18	68	64	13	80	57	0.041	PCR-RFLP
Rafrafi	2013	Tunisia	African	lung cancer	170	225	PB	25	67	78	30	95	100	0.329	PCR-RFLP
Wang	2014	China	Asian	hepatocellular carcinoma	205	208	HB	20	105	80	36	96	76	0.549	PCR-RFLP/PCR-SSP
Koensgen	2014	Germany	Caucasian	ovarian cancer	267	426	HB	69	150	48	72	226	128	0.1	PCR-RFLP
Chen	2016	China	Asian	osteosarcoma	190	190	HB	14	90	86	12	88	90	0.116	PCR-RFLP
Taheri	2019	Iran	Asian	prostate cancer	355	200	HB	61	170	124	32	92	76	0.639	ARMS-PCR
Kaanane	2022	Morocco	African	lung cancer	150	150	PB	20	40	90	8	44	98	0.307	TaqMan
Alkanli	2023	Turkey	Caucasian	bladder cancer	88	89	HB	14	46	28	13	45	31	0.608	PCR-RFLP
Moreno-Guerrero	2021	Mexico	Mixed	neuroblastoma	27	38	HB	3	16	8	16	15	7	0.313	PCR-RFLP
Song	2009	China	Asian	gastric cancer	125	140	HB	27	58	40	24	63	53	0.48	PCR-RFLP
Fu	2016	China	Asian	glioma	127	284	HB	18	55	54	27	118	139	0.788	PCR-RFLP
Zhang	2017	China	Asian	breast cancer	442	447	HB	18	225	199	40	202	205	0.327	PCR-RFLP
Huang	2018	China	Asian	nasopharyngeal carcinoma	176	352	HB	23	74	79	56	164	132	0.671	PCR-RFLP
Ghazy	2021	Saudi Arabia	Asian	prostate cancer	40	40	HB	11	17	12	22	16	2	0.673	real-time PCR
Liao	2011	China	Asian	hepatocellular carcinoma	150	150	HB	18	68	64	13	80	57	0.041	PCR-RFLP
Elsamanoudy	2015	Egypt	African	hepatocellular carcinoma	112	105	HB	8	36	68	24	45	36	0.178	PCR-RFLP
Qin	2012	China	Asian	hepatocellular carcinoma	150	150	HB	18	68	64	13	80	57	0.041	PCR-RFLP
Savage	2006	USA	Caucasian	gastric cancer	288	428	PB	68	140	80	91	204	133	0.438	TaqMan
Lu	2015	China	Asian	hepatocellular carcinoma	454	446	HB	43	236	175	61	183	202	0.062	PCR-RFLP

*HB* Hospital-based, *PB* Population-based, *SOC* Source of control, *PCR-RFLP* Polymerase chain reaction followed by restriction fragment length polymorphism, *SSP* Sequence specific primer, *AS* Allele specific primer, *ARMS* Amplification refractory mutation system, *HWE* Hardy-Weinberg equilibrium of control group

### Pooled analyses

**Fig. 2:**
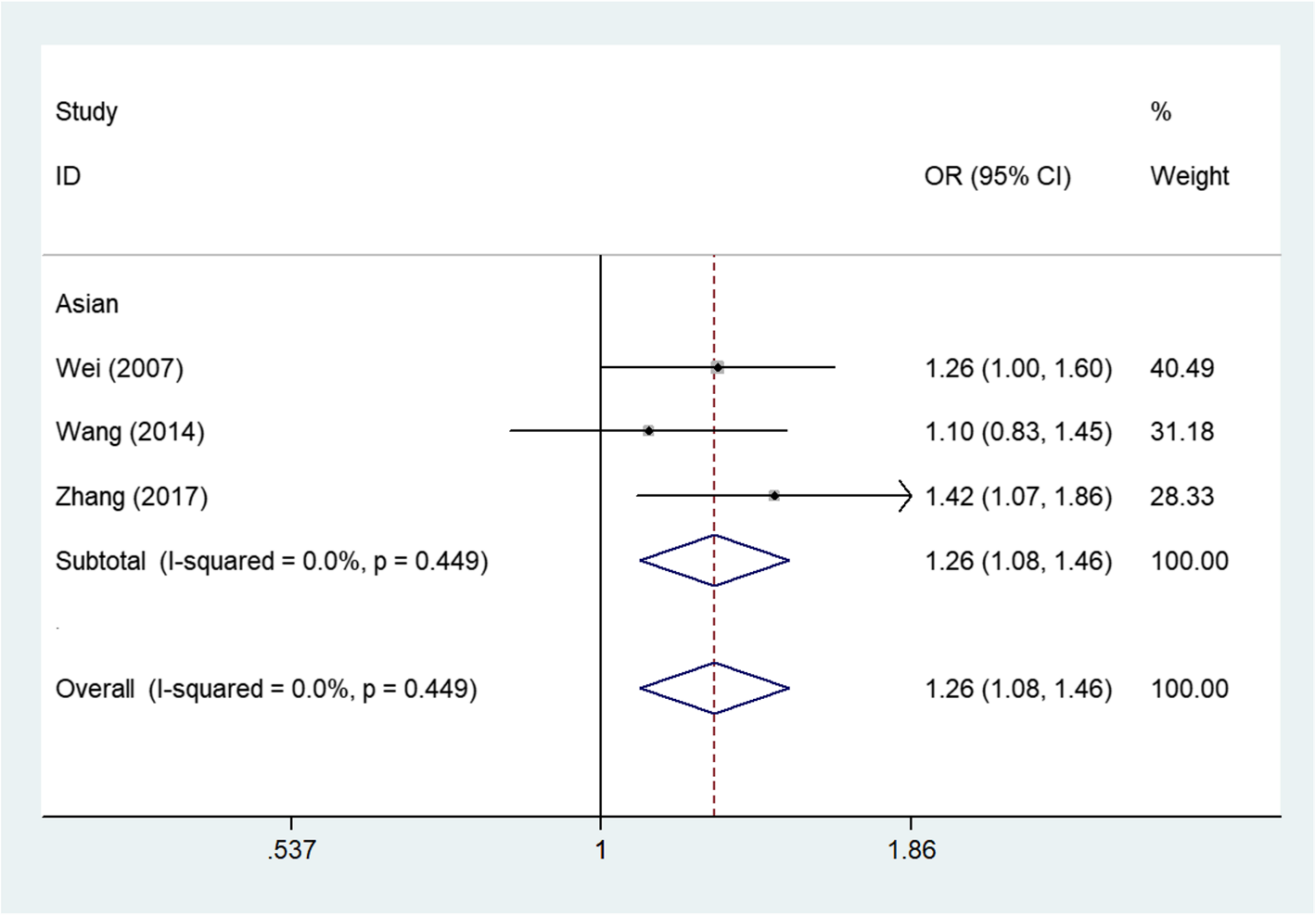
Forest plots corresponding to cancer-related risk when assessing the relationship between the CXCL8 -353 polymorphism (AA + AT vs. TT) in all cancers. The squares and horizontal lines respectively correspond to the study-specific ORs and 95% CIs, with square area being indicative of weight (the inverse of the variance). Diamonds additionally reflect the summary OR and 95% CI

The results of pooled analyses pertaining to the *CXCL8* -353 polymorphism are presented in Table 2. A significant increase in the association between this polymorphism and cancer risk was detected under four genetic models: OR = 1.255, 95%CI (1.079–1.459), *P*_heterogeneity_ = 0.449, *P* = 0.003 for A-allele vs. T-allele, Fig. 2; OR = 1.463, 95%CI (1.068–2.004), *P*_heterogeneity_ = 0.653, *P* = 0.018 for AA vs. TT; OR = 1.339, 95%CI (1.052–1.705), *P*_heterogeneity_ = 0.524, *P* = 0.018 for AA + AT vs. TT; OR = 1.297, 95%CI (1.031–1.632), *P*_heterogeneity_ = 0.784, *P* = 0.026 for AA vs. AT + TT.

**Table 2:** Stratified subgroups analyses for CXCL8 genes polymorphisms and cancer susceptibility

Variables	No	Case/Controls	M-allele vs. W-allele			MM vs. WW			MW vs. WW			MM + MW vs. WW			MM vs. MW + WW		
			OR(95%CI)	*P*_h_	*P*	OR(95%CI)	*P*_h_	*P*	OR(95%CI)	*P*_h_	*P*	OR(95%CI)	*P*_h_	*P*	OR(95%CI)	*P*_h_	*P*
CXCL8 -353 (rs1454941)	3	927/945	**1.255(1.079–1.459)**	**0.449**	**0.003**	**1.463(1.068–2.004)**	**0.653**	**0.018**	1.269(0.980–1.643)	0.732	0.070	**1.339(1.052–1.705)**	**0.524**	**0.018**	**1.297(1.031–1.632)**	**0.784**	**0.026**
CXCL8 +678 (rs7374124)	3	690/768	1.020(0.866–1.201)	0.970	0.816	1.166(0.837–1.623)	0.814	0.364	0.881(0.696–1.115)	0.390	0.291	0.952(0.770–1.178)	0.785	0.652	1.203(0.875–1.655)	0.702	0.256
CXCL8 +1633 (rs2227543)	4	823/1104	0.968(0.804–1.166)	0.166	0.733	0.937(0.651–1.349)	0.197	0.727	0.978(0.789–1.211)	0.569	0.837	0.963(0.769–1.206)	0.304	0.740	0.935(0.719–1.215)	0.372	0.614
CXCL8 +2767 (rs1126647)	5	1007/1624	0.930(0.799–1.082)	0.149	0.347	0.875(0.626–1.224)	0.094	0.435	0.924(0.774–1.102)	0.577	0.380	0.913(0.774–1.078)	0.532	0.283	0.905(0.638–1.283)	0.034	0.575
CXCL8 +781 (rs2227306)																	
Total	24	4686/5606	0.952(0.850–1.066)	0.000	0.395	0.976(0.860–1.109)	0.003	0.712	0.923(0.719–1.183)	0.000	0.526	0.965(0.840–1.108)	0.000	0.610	0.930(0.755–1.147)	0.000	0.499
Ethnicity																	
Asian	16	3473/3937	0.948(0.854–1.054)	0.005	0.323	0.884(0.692–1.131)	0.003	0.327	0.969(0.841–1.117)	0.021	0.667	0.956(0.833–1.098)	0.013	0.523	0.893(0.715–1.116)	0.004	0.321
Caucasian	4	754/1151	1.180(0.947–1.469)	0.063	0.139	1.239(0.998–1.537)	0.126	0.056	1.459(0.912–2.334)	0.063	0.115	1.255(0.883–1.786)	0.046	0.206	**1.320(1.046–1.666)**	**0.375**	**0.019**
African	3	432/480	0.826(0.406–1.681)	0.000	0.598	0.809(0.199–3.297)	0.000	0.767	0.746(0.461–1.205)	0.070	0.231	0.750(0.370–1.521)	0.001	0.426	0.932(0.282–3.077)	0.001	0.907
Cancer type																	
Gastric cancer	4	732/966	1.120(0.974–1.286)	0.666	0.111	1.127(0.906–1.402)	0.618	0.284	1.255(0.947–1.664)	0.852	0.114	1.158(0.943–1.422)	0.571	0.162	1.158(0.903–1.486)	0.958	0.248
Hepatocellular carcinoma	6	1202/1399	0.778(0.595–1.019)	0.000	0.068	0.602(0.357–1.017)	0.004	0.058	0.826(0.575–1.187)	0.001	0.301	0.763(0.529–1.100)	0.000	0.147	0.659(0.415–1.045)	0.011	0.076
Prostate cancer	2	395/240	0.613(0.183–2.056)	0.001	0.428	0.352(0.026–4.717)	0.003	0.431	0.535(0.089–3.207)	0.031	0.494	0.429(0.048–3.804)	0.006	0.447	0.622(0.183–2.114)	0.018	0.447
Oral cancer	2	420/500	0.877(0.724–1.063)	0.470	0.181	0.866(0.548–1.368)	0.289	0.536	0.765(0.579–1.012)	0.956	0.061	0.783(0.600–1.021)	0.792	0.070	1.014(0.627–1.639)	0.247	0.955
Lung cancer	2	320/375	1.181(0.819–1.704)	0.127	0.373	1.617(0.650–4.022)	0.083	0.302	0.939(0.674–1.306)	0.791	0.707	1.065(0.785–1.443)	0.363	0.687	1.650(0.694–3.924)	0.089	0.257
Glioma	2	427/584	1.117(0.883–1.414)	0.235	0.355	1.343(0.888–2.031)	0.366	0.163	0.990(0.728–1.346)	0.270	0.949	1.069(0.780–1.463)	0.228	0.680	1.346(0.906–2.002)	0.543	0.142
Other cancer	6	1190/1542	0.976(0.747–1.274)	0.000	0.856	0.858(0.426–1.727)	0.000	0.667	0.980(0.868–1.107)	0.096	0.358	1.080(0.790–1.477)	0.009	0.629	0.801(0.451–1.422)	0.000	0.448
Source of control																	
HB	20	3967/4595	0.920(0.806–1.050)	0.000	0.215	0.849(0.635–1.135)	0.000	0.268	0.971(0.834–1.130)	0.001	0.701	0.942(0.798–1.110)	0.000	0.474	0.863(0.678–1.099)	0.000	0.232
PB	4	719/1011	1.096(0.951–1.264)	0.336	0.207	0.992(0.800–1.231)	0.765	0.944	1.274(0.950–1.709)	0.279	0.106	1.063(0.869–1.301)	0.597	0.550	1.234(0.948–1.607)	0.279	0.118

*P*_*h*_ Value of *Q*-test for heterogeneity test, *P Z*-test for the statistical significance of the OR, *HB* Hospital-based, *PB* Population-based, *M* Mutation, *W* Wild

**Fig. 3:**
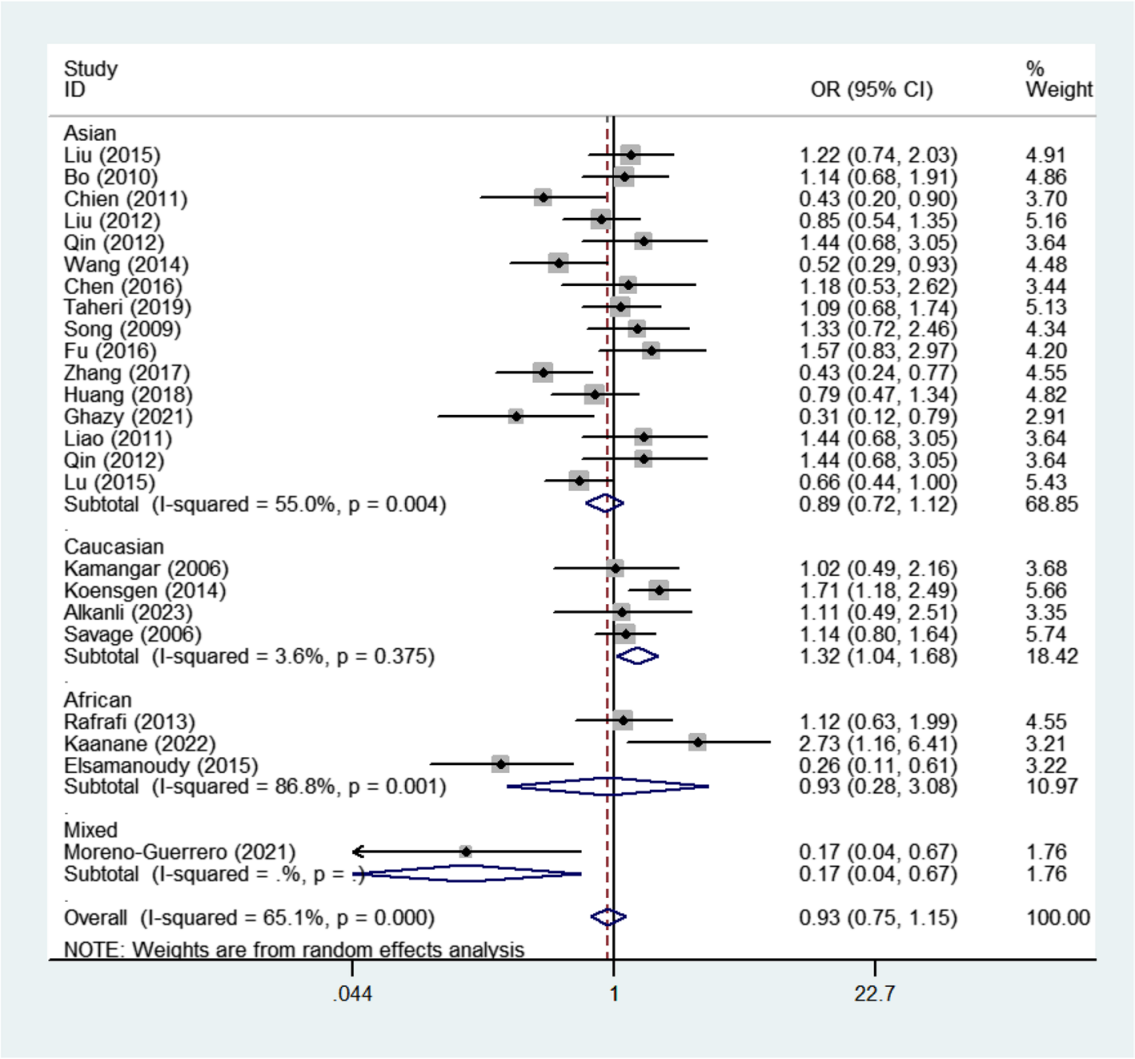
Forest plots corresponding to the association between the CXCL8 +781 polymorphism (TT vs. TC + CC) and cancer risk according to ethnicity

Pooled analyses focused on the *CXCL8* + 781 polymorphism failed to detect any significant association with overall cancer risk, and the same was true when conducting subgroup analyses based on cancer type or the source of control subjects (Table 2). However, ethnicity-based subgroup analyses revealed an increase in risk associated with the + 781 polymorphism among Caucasians [TT vs. TC + CC, OR = 1.320, 95%CI (1.046–1.666), *P*_heterogeneity_ = 0.375, P = 0.019, Fig. 3] (Table 2).

For the three other *CXCL8* polymorphisms (+678, +1633, +2767), no significant associations with overall cancer risk were detected for different variant genotypes under the analyzed genetic models (Table 2).

### Publication bias analyses

The potential for publication bias was next evaluated with Begg’s funnel plots and Egger’s test. Funnel plots appeared to exhibit some asymmetry, suggesting some potential bias (+2767 and +781 polymorphisms) with respect to allele comparisons for the selected *CXCL8* polymorphisms (Supplementary Table 1). Egger’s test confirmed this evidence of publication bias (Supplementary Table 1). To further access the publication bias, Trim and fill model was applied if publication bias was detected by Egger’s test. Finally, +2767 polymorphism was no longer found publication bias in the recessive (MM vs. MW + WW) genetic model (Supplementary Figure 1A). However, publication bias remains from +781 polymorphism in three genetic models (Supplementary Figure 1B-D).

### Big data analytics and our own clinical analysis for oral cancer

**Fig. 4:**
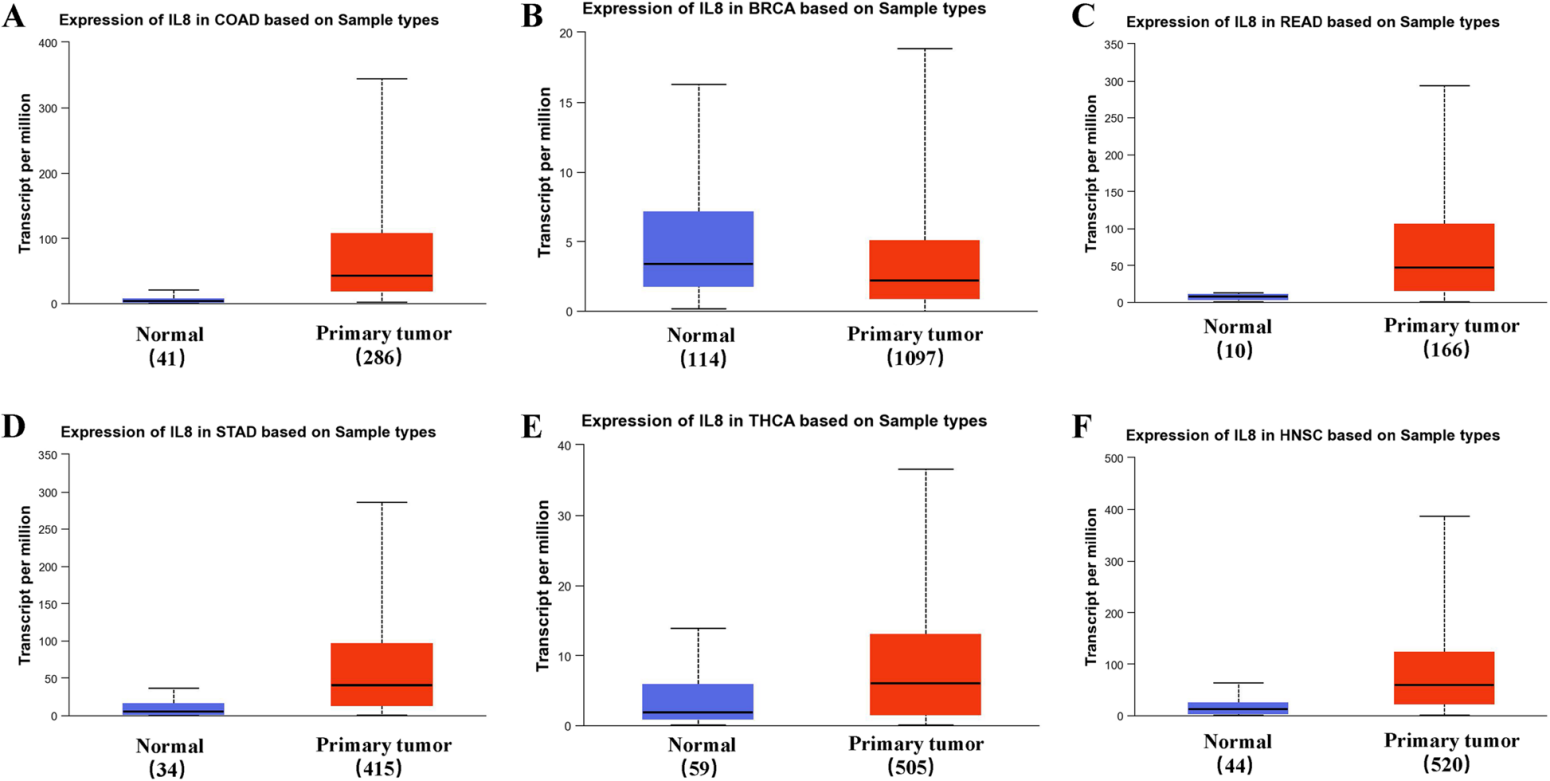
CXCL8 expression in tumors and normal tissues for six cancer types (https://ualcan.path.uab.edu/analysis.html). **A** CXCL8 expression in colon adenocarcinoma (COAD) was elevated relative to normal tissues (*P* < 0.01). **B** CXCL8 expression in urothelial carcinoma (URCA) was reduced relative to normal tissues (*P* < 0.01). **C**-**F** CXCL8 expression levels were elevated in tumor tissues as compared to healthy control samples in rectal adenocarcinoma (READ) (**C**), stomach adenocarcinoma (STAD) (**D**), thyroid carcinoma (THCA) (**E**), and head and neck squamous cell carcinoma (HNSC) (**F**) (All *P* < 0.01)

**Fig. 5:**
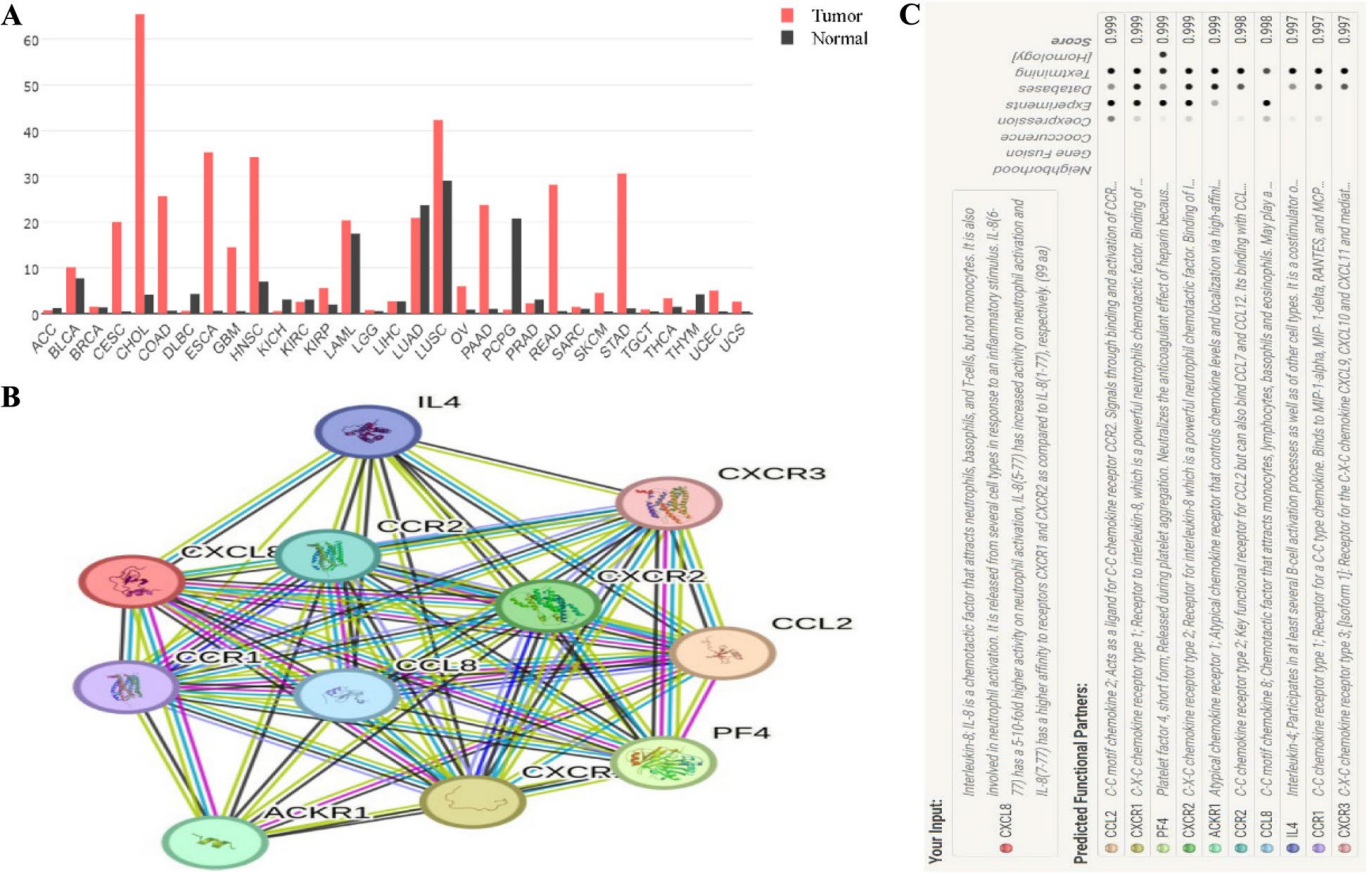
**A** CXCL8 expression levels in tumors and control tissues were compared with the GEPIA database. **B** Visualization of interacting proteins associated with CXCL8. **C** Interacting protein scores for proteins associated with CXCL8. ACC, adrenocortical carcinoma; BRCA, breast invasive carcinoma; CHOL, cholangiocarcinoma; DLBC, lymphoid neoplasm diffuse large B-cell lymphoma; GBM, glioblastoma multiforme; KICH, kidney chromophobe; KIRP, kidney renal papillary cell carcinoma; LGG, brain lower grade glioma; LUAD, lung adenocarcinoma; OV, ovarian serous cystadenocarcinoma; PCPG, pheochromocytoma and paraganglioma; READ, rectum adenocarcinoma; SKCM, skin cutaneous melanoma; TGCT, testicular germ cell tumors; THYM, thymoma; UCS, uterine carcinosarcoma

To better explore *CXCL8* expression in tumor tissue samples, the UALCAN database was next utilized, revealing that relative to corresponding normal tissue controls, *CXCL8* levels in tumors were elevated in colon adenocarcinoma (*P* < 0.01), rectum adenocarcinoma (*P* < 0.01), stomach adenocarcinoma (*P* < 0.01), thyroid carcinoma (*P* < 0.01), and head and neck squamous cell carcinoma (*P* < 0.01), whereas it was downregulated in bladder urothelial carcinoma as compared to tumor tissues (*P* < 0.01) (Fig. 4). These trends toward altered *CXCL8* expression were further confirmed with the GEPIA database. To better understand the functions of CXCL8, the STRING database was leveraged to identify the 10 proteins that most closely interact with CXCL8 (Fig. 5).

**Fig. 6:**
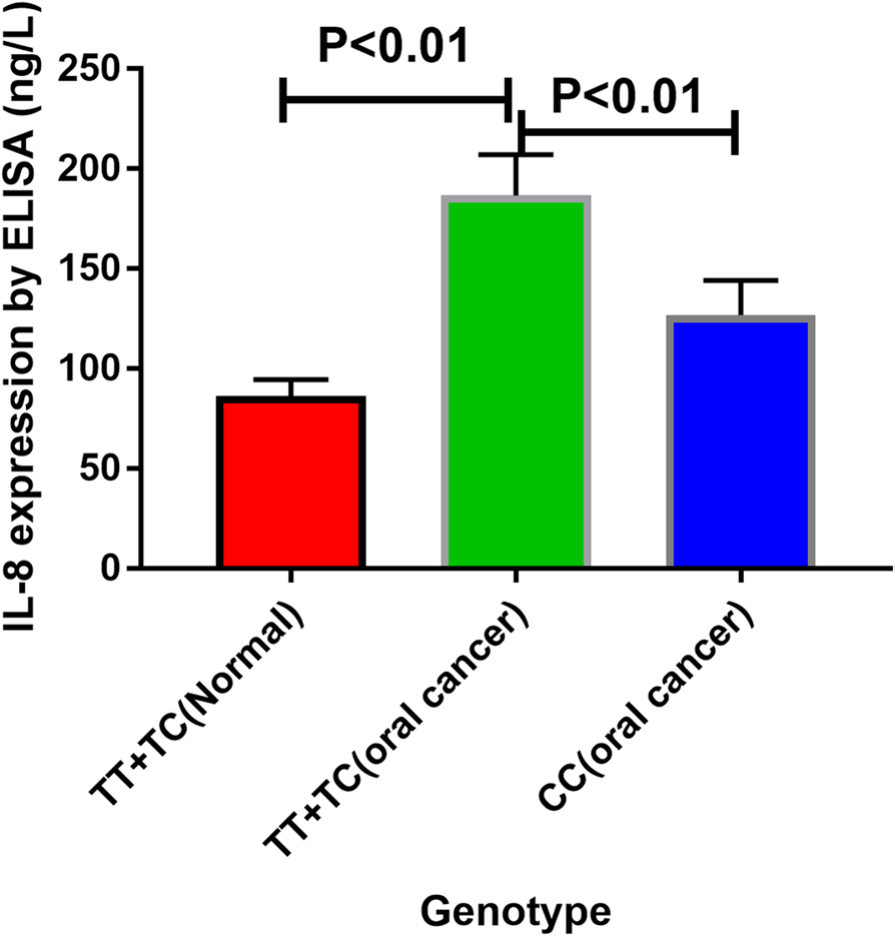
ELISA analyses of serum CXCL8 levels in oral cancer patients with different +781 genotypes (horizontal lines, mean values). Higher serum CXCL8 concentrations were detected in patients with oral cancer harboring the TT + TC genotypes as compared to those with the CC genotype (*P* < 0.01). Serum CXCL8 concentrations were also significantly higher in oral cancer patients with the TT + TC genotypes as compared to healthy controls with the same genotypes (*P* < 0.01)

Given the upregulation of CXCL8 in most tumor tissues and the close association between cancer risk and two *CXCL8* gene polymorphisms, the impact of different polymorphic loci on the production of CXCL8 was next assessed in oral cancer patients and healthy controls. Ultimately this approach revealed that serum CXCL8 concentrations were significantly higher in oral cancer patients harboring the TT + TC genotypes as compared to the CC genotype (*P* < 0.01). Serum CXCL8 levels in oral cancer patients with the TT + TC genotypes were also significantly elevated as compared to levels in normal control subjects (*P* < 0.01) (Fig. 6).

## Discussion

Single nucleotide polymorphisms (SNPs) are the most common type of genetic variant present within the human genome. Point polymorphisms with minor alleles of at least 1% in at least one population are regarded as SNPs [38]. SNPs that present within regulatory regions have the potential to impact transcriptional activity, while SNPs located in 3’-untranslated regions may impact the stability of the encoded RNA, SNPs in splice sites can affect splicing activity, and SNPs within coding regions can impact the sequence of the final protein. Different SNPs are believed to be key contributors to variations among individuals with respect to susceptibility to cancer and other diseases [39].

This meta-analysis is the first publication to have conducted a systematic evaluation of the relationship between five CXCL8 polymorphisms and overall cancer risk. This pooled analysis incorporated a total of 7845 cases and 9619 controls, including 927 cases and 945 controls pertaining to the -353 SNP, 690 cases and 768 controls related to the +678 SNP, 823 cases and 1104 controls related to the +1633 SNP, 1007 cases and 1624 controls related to the +2767 SNP, and 4686 cases and 5606 controls related to the +781 SNP. Finally, no significant association was found among +678, +1633, +2767 polymorphisms and cancer risk, based on current limited samples, further larger sample research should be carried out. In pooled analyses, a significant link between the -353 polymorphism and an elevated risk of cancer was noted, while the +781 polymorphism was specifically associated with greater cancer risk among Caucasians although this same association was not detected in African or Asian populations. We surmised that -353 or +781 polymorphism may increase the expression of CXCL8, as a similar oncogene, which can result in the increased incidence of cancer.

The existence of SNP in target regions of miRNAs could result in the regulation and alteration of gene expression that are the critical points in the pathogenicity of diseases. Based on solid evidence, the occurrence of single-nucleotide variation in miRNA binding sites via alteration in the binding affinity to SNP sites and post-transcriptional dysregulations could affect carcinogenesis risk, survival score, and cancer invasion [40–42]. For example, Kaiyan Dong et al. found that T-allele, CT, and CT + TT genotypes of rs3748067 adjusted for drinking status, smoking habits, and family history of gastric cancer are associated with a significant reduction in the gastric carcinogenesis risk [43]. In addition, both miR146a rs2910164 and miR499a rs3746444 can influence the expression of CXCL8 and were associated with the development of cutaneous leishmaniasis caused by leishmania guyanensis [44]. Furthermore, Kaviani et al. suggested CXCL8 was involved in key molecular mechanisms related to the promotion of inflammation and oxidative stress and subsequently the development of gastric cancer, and also was considered as cut-point druggable protein, which maybe the potential of targeting for therapeutic objective [45]. Above articles indicated SNPs from CXCL8 may be associated with the different expression of CXCL8 and status of inflammation and oxidative stress, then result in the development of cancer and be considered as a druggable protein for treatment of cancer.

These findings may be influenced by a range of variables. For one, differences in ethnicity distributions in the case and control groups may have confounded the pooled analyses. Cancers are also complex multifactorial diseases such that both genetic and environmental factors ultimately shape disease onset and progression, with no single factor having a major effect on disease susceptibility in many cases [46]. Exposure to carcinogenic risk factors including radiation, infectious agents, dietary factors, and tobacco smoke can all raise the risk of oncogenesis, but precisely quantifying the magnitude of the risk associated with these exposures can be challenging. Lastly, the specific polymorphic sites within the *CXCL8* gene can have varied functional effects, leading to distinct changes in CXCL8 expression that may ultimately translate to shifts in the risk of developing cancer.

Intensive research efforts in recent years have focused on developing approaches to detecting tumors during their earlier stages of development, providing a means of improving survival outcomes for affected patients. Early detection strategies can improve the efficacy of surgery and other interventional approaches while mitigating the economic and psychological burden associated with an advanced disease diagnosis. It is thus essential that easy-to-use, minimally invasive, cost-effective technologies be developed capable of detecting tumors when they are precancerous lesions or remain in the early stages of disease [47]. The present results revealed that CXCL8 expression was elevated in most surveyed tumor types as compared to corresponding normal tissues. Higher CXCL8 levels were also observed in the serum of oral cancer patients with the T-allele or TT genotype, suggesting this may offer value as a biomarker suitable for use when detecting oral cancer. Future studies may be able to apply these results to guide diagnostic and therapeutic approaches aimed at abrogating cancer-related risk.

Cancer develops in progresses in a manner that is complex and driven by a wide range of interacting factors. As such, efforts focused solely on a single polymorphism are inherently limited. As such, the STRING database was leveraged to identify other related genes that may be related to oncogenic risk. Among the 10 most closely associated proteins identified in this analysis, the *CCL2* -2518A/G polymorphism is reportedly closely related to the risk of gynecological cancer [48]. Moreover, the *CCR2* -V64I polymorphism is potentially associated with the incidence of cancers including oral, cervical, and bladder cancers [49], while *IL4* rs2243250 and rs79071878 have been linked to oncogenesis in certain cancers and ethnic groups [50]. In light of these analyses, further in-depth studies focused on these CXCL8-related genes and gene-gene interactions are warranted in order to better guide efforts to treat oral cancer and other malignancies.

This study is subject to multiple limitations. For one, although all relevant articles were incorporated into the present meta-analysis, the overall sample size remained relatively small, and these numbers were further reduced when stratifying studies according to ethnicity, cancer type, or source of controls. There were also relatively few case-control studies focused on the +678, +1633, +2767, or -353 polymorphisms were also limited. May be studies with huge number of samples are needed to assess it in the future. Secondly, the risk of cancer in patients harboring these polymorphisms may be influenced by gene-gene, gene-environment, and other polymorphic interactions. This meta-analysis was also performed based upon estimates that were not adjusted, and future efforts to obtain details pertaining to patient age, sex, and tumor staging may permit more granular and precise analyses. Thirdly, we re-reviewed all included studies, because many kinds of cancer were analyzed, however, different standards about tumor stage existed, so we can’t merger together. The limited of the number of included studies is also the cause that we also can’t analyze the subgroup for like age, sex, smoking, drinking, and so on. Some publication bias was found in two polymorphisms (+2767 and +781 polymorphisms), which indicated that heterogeneity was existed in included studies. Further studies should avoid above limitation. Lastly, the overall results of this study are not representative of all cancer types, as only certain cancers were available for analysis and the number of patients varied markedly among cancer types. Some significant polymorphisms of CXCL8 may have some potential clinical applications: such as some related inhibitors.

In conclusion, the results of the present meta-analysis support a potential link between the *CXCL8* -353 and +781 polymorphisms and an overall increase in cancer risk in the general population or in individuals of particular ethnicities. The +781 polymorphism was additionally established as a potential diagnostic biomarker for oral cancer. Even so, further large-scale studies with more substantial sample sizes and simultaneous analyses of multiple SNPs in one or more CXCL8 polymorphisms will be essential to reliably clarify the CXCL8-specific genetic antecedent of solid tumor development.

### Supplementary Information

Supplementary Material 1.

## Data Availability

The datasets used and/or analyzed during the current study are available from the corresponding author on reasonable request.
