# Supplementary material for: Meta-analyses of the relationship between five CXCL8 gene polymorphisms and overall cancer risk, and a case-control study of oral cancer
Source: BMC Oral Health. 2024 May 28;24:622. doi: 10.1186/s12903-024-04330-6 (PMC11131276; doi:10.1186/s12903-024-04330-6)
Supplement: Supplementary file 1 — Supplementary Material 1. [file 12903_2024_4330_MOESM1_ESM.docx]

**Supplementary Table 1** Publication bias tests (Begg’s funnel plot and Egger’s test for publication bias test) for *CXCL8* gene five polymorphisms (+781, -353, +678, +1633, +2767).

| Egger's test |  |  |  |  |  |  | | Begg's test | |  |
| --- | --- | --- | --- | --- | --- | --- | --- | --- | --- | --- |
| Genetic type | Coefficient | Standard error | *t* | *P*-value | 95%CI of intercept |  | | *z* | | *P* value |
| -353 |  |  |  |  |  |  | |  | |  |
| M-allele vs. W-allele | -1.075 | 9.397 | -0.11 | 0.927 | (-120.487- 118.336) | | 0 | | 1 | |
| MW vs. WW | 0.793 | 1.435 | 0.55 | 0.679 | (-17.441- 19.027) |  | | 0 | | 1 |
| MM vs. WW | 0.797 | 3.121 | 0.26 | 0.841 | (-38.859- 40.453) |  | | 0 | | 1 |
| MM+MW vs. WW | 0.926 | 1.641 | 0.56 | 0.673 | (-19.923- 21.776) |  | | 0 | | 1 |
| MM vs. MW+WW | -2.476 | 0.331 | -7.84 | 0.085 | (-6.681- 1.728) |  | | 0 | | 1 |
| +678 |  |  |  |  |  |  | |  | |  |
| M-allele vs. W-allele | -1.167 | 0.635 | -1.84 | 0.317 | (-9.245- 6.911) |  | | 0 | | 1 |
| MW vs. WW | 0.689 | 0.453 | -1.52 | 0.371 | (-6.456- 5.078) |  | | 0 | | 1 |
| MM vs. WW | -0.831 | 0.157 | -5.26 | 0.12 | (-2.838- 1.176) |  | | 0 | | 1 |
| MM+MW vs. WW | -1.191 | 0.338 | -3.52 | 0.176 | (-5.486- 3.104) |  | | 0 | | 1 |
| MM vs. MW+WW | 0.745 | 0.262 | -2.84 | 0.215 | (-4.079- 2.588) |  | | 0 | | 1 |
| +1633 |  |  |  |  |  |  | |  | |  |
| M-allele vs. W-allele | 2.559 | 3.776 | 0.68 | 0.568 | (-13.687- 18.805) |  | | 0.34 | | 0.734 |
| MW vs. WW | 1.963 | 2.197 | 0.89 | 0.466 | (-7.489- 11.417) |  | | 0.34 | | 0.734 |
| MM vs. WW | 0.929 | 1.786 | 0.52 | 0.655 | (-6.755- 8.615) |  | | 0.34 | | 0.734 |
| MM+MW vs. WW | 2.025 | 2.316 | 0.87 | 0.474 | (-7.941- 11.991) |  | | 0.34 | | 0.734 |
| MM vs. MW+WW | 0.822 | 2.053 | 0.4 | 0.727 | (-8.014- 9.66) |  | | 0.34 | | 0.734 |
| +2767 |  |  |  |  |  |  | |  | |  |
| M-allele vs. W-allele | 6.931 | 2.409 | -2.88 | 0.064 | (-14.598- 0.736) |  | | 1.22 | | 0.221 |
| MW vs. WW | -4.559 | 2.293 | -1.99 | 0.141 | (-11.858- 2.740) |  | | 1.22 | | 0.221 |
| MM vs. WW | -7.603 | 2.546 | -2.99 | 0.058 | (-15.707- 0.500) |  | | 1.22 | | 0.221 |
| MM+MW vs. WW | -5.046 | 2.506 | -2.01 | 0.137 | (-13.021- 2.927) |  | | 1.22 | | 0.221 |
| MM vs. MW+WW | -7.780 | 2.335 | -3.33 | 0.045 | (-15.214- 0.346) |  | | 1.71 | | 0.086 |
| +781 |  |  |  |  |  |  | |  | |  |
| M-allele vs. W-allele | -1.891 | 0.754 | -2.51 | 0.020 | (-3.456- 0.327) |  | | 1.29 | | 0.197 |
| MW vs. WW | -0.885 | 0.550 | -1.61 | 0.122 | (-2.025- 0.256) |  | | 0.94 | | 0.346 |
| MM vs. WW | -0.988 | 0.357 | -2.77 | 0.011 | (-1.729- 0.248) |  | | 1.59 | | 0.112 |
| MM+MW vs. WW | -0.932 | 0.593 | -1.57 | 0.130 | (-2.162- 0.297) |  | | 0.79 | | 0.427 |
| MM vs. MW+WW | -1.284 | 0.408 | -3.15 | 0.005 | (-1.131- 0.437) |  | | 1.64 | | 0.102 |

M: mutation; W: wild

**Supplementary Figure 1** The publication bias and make asymmetry funnel plot to symmetrical using various techniques like Trim and fill model.


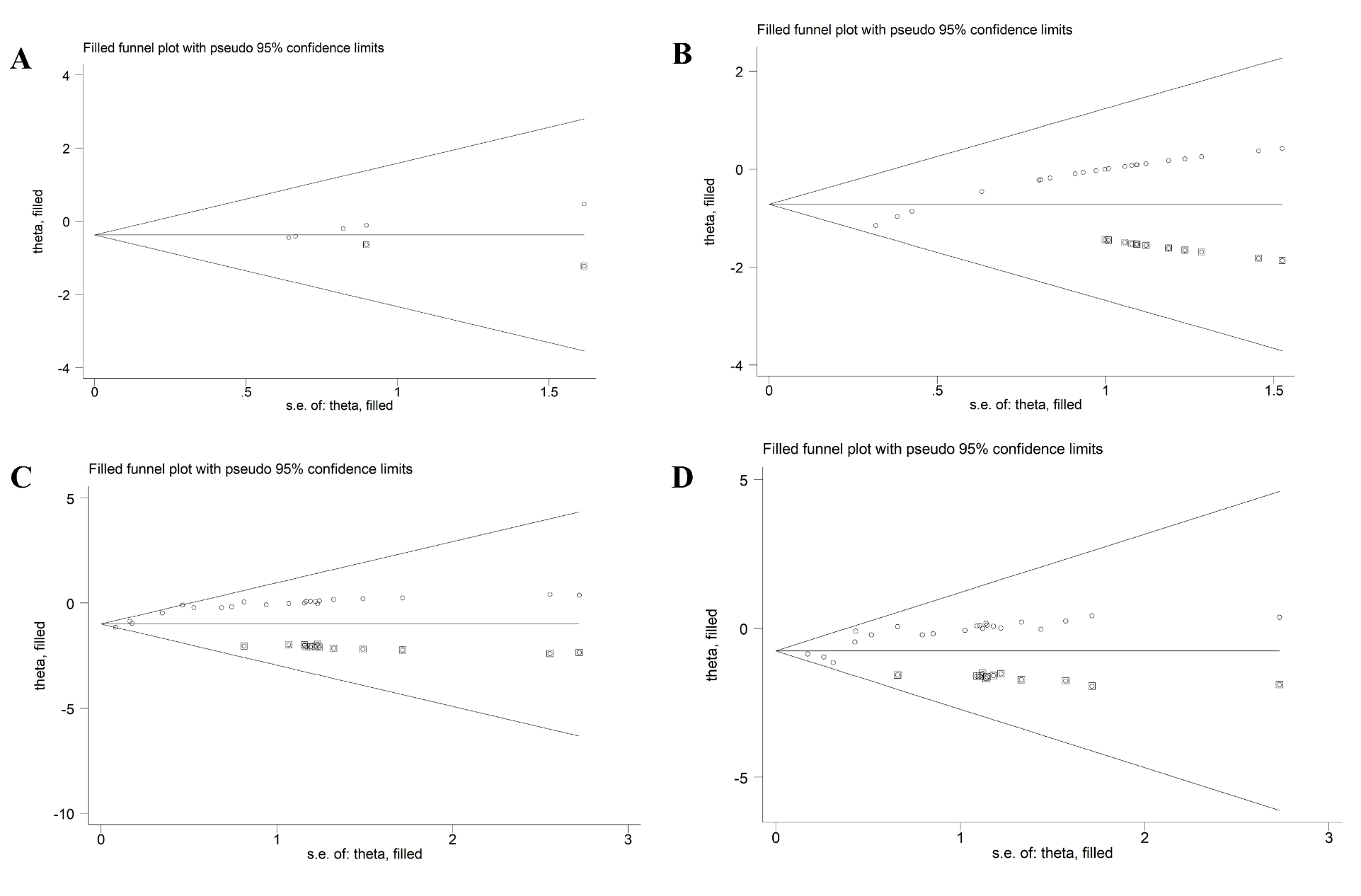

A:+2767 polymorphism in the recessive (MM vs. MW+WW) genetic model. B: +781 polymorphism in the recessive (M vs. W) genetic model. C: +781 polymorphism in the recessive (MM vs. WW) genetic model. D: +781 polymorphism in the recessive (MM vs. MW+WW) genetic model.
